# Links between the genetic determinants of morning plasma cortisol and body shape: a two-sample Mendelian randomisation study

**DOI:** 10.1038/s41598-024-53727-4

**Published:** 2024-02-08

**Authors:** Sofia Christakoudi, Alexandros-Georgios Asimakopoulos, Elio Riboli, Konstantinos K. Tsilidis

**Affiliations:** 1 Department of Epidemiology and Biostatistics, School of Public Health, Imperial College London, White City Campus, 90 Wood Lane, London, W12 0BZ UK; 2 Department of Hygiene and Epidemiology, University of Ioannina School of Medicine, Ioannina, Greece

**Keywords:** Cortisol, Body shape, Obesity, Mendelian randomisation, Obesity, Adrenal gland diseases, Epidemiology, Genetics research

## Abstract

High cortisol production in Cushing’s syndrome leads to fat centralisation. The influence of modest cortisol variations on body shape, however, is less clear. We examined potentially causal associations between morning plasma cortisol and body shape and obesity with inverse-variance weighted random-effects models in a two-sample Mendelian randomisation analysis. We used publicly available summary statistics from the CORtisol NETwork (CORNET) consortium, UK Biobank, and the Genetic Investigation of Anthropometric Traits (GIANT) consortium. Only in women, morning plasma cortisol (proxied by ten genetic polymorphisms) was associated positively with waist size reflected in waist-to-hip index (WHI, 0.035 standard deviation (SD) units change per one SD cortisol increase; 95% confidence interval (0.002–0.067); p = 0.036) and “a body shape index” (ABSI; 0.039 (0.006–0.071); p = 0.021). There was no evidence for associations with hip index (HI) or body mass index (BMI). Among individual polymorphisms, *rs7450600* stood out (chromosome 6; Long Intergenic Non-Protein-Coding RNA 473 gene, *LINC00473*). Morning plasma cortisol proxied by *rs7450600* was associated positively with WHI and inversely with HI and BMI in women and men. Our findings support a causal association of higher morning plasma cortisol with larger waist size in women and highlight *LINC00473* as a genetic link between morning plasma cortisol and body shape.

## Introduction

Cortisol is an adrenal glucocorticoid hormone and plays a key role in the regulation of energy expenditure, fat distribution, and lipid metabolism^1^. It is well known that a prolonged exposure to very high glucocorticoid levels, as in Cushing’s syndrome, leads to fat centralisation and development of abdominal obesity^2^, which are reversed after adrenalectomy^3^. More modest cortisol increases, as in functional adrenal incidentalomas with autonomous cortisol production, similarly lead to larger visceral fat depots^4^. It is less clear, however, whether more modest variations of cortisol levels can alter body shape and contribute to the development of abdominal obesity^5^. Investigating the determinants of body shape is important because fat depots are differentially associated with cardiometabolic conditions, positively for visceral fat accumulation but inversely for gluteofemoral fat accumulation^6^. Although cross-sectional observational studies have shown that individuals with abdominal obesity have higher free cortisol excretion and higher responsiveness to stimulation with corticotropin releasing hormone (CRH) compared to individuals with peripheral fat distribution^7^, confirming a causal relationship would require prospective interventional studies, which are harder to perform. Mendelian randomisation (MR) analysis is a more practical approach, which uses single nucleotide polymorphisms (SNPs) as instrumental variables (IVs) and permits the interpretation of associations between the genetically predicted exposure and the outcome as potentially causal, conditional on a set of assumptions^8^.

The main genetic region associated with the inter-individual variability of morning plasma cortisol was identified in a genome-wide association meta-analysis (GWAMA) by the CORtisol NETwork (CORNET) consortium and includes the *SERPINA6* gene (coding corticosteroid binding globulin (CBG), the main cortisol carrier protein) and *SERPINA1* gene (coding α1-antitrypsin, which inhibits the cleavage and inactivation of CBG by neutrophil elastase)^9^. A more recently updated GWAMA has confirmed the importance of the *SERPINA6/SERPINA1* locus but could not identify further loci with genome-wide significance, despite doubling the sample size and tripling the number of genetic polymorphisms^10^. Although mutations in *SERPINA6* have been associated with attenuated clinical features of cortisol deficit^11^ and common genetic polymorphisms in the *SERPINA6* locus have been associated with altered cortisol release^10^, it cannot be assumed that all polymorphisms related to CBG levels in blood would additionally affect cortisol release. Hence, there is a need to diversify the genetic instrument for cortisol and to include genetic variants outside the *SERPINA6* locus.

Based on IVs derived from the first GWAMA^9^, an MR study has shown inverse associations between genetically predicted morning plasma cortisol and general obesity class I^12^. These findings, however, could not be confirmed using body mass index (BMI) on a continuous scale and IVs for cortisol identified in the updated GWAMA^10^. No study, to our knowledge, has so far used MR to examine potentially causal associations of cortisol with body shape. Moreover, waist and hip circumferences and the waist-to-hip ratio (WHR), which have traditionally been used as indices of body shape, are correlated strongly positively with BMI and thus reflect general obesity in addition to abdominal obesity and body shape^13^. The allometric “a body shape index” (ABSI), on the other hand, reflects waist circumference among individuals with the same weight and height and is thus uncorrelated with BMI^14^. In analogy to ABSI, hip index (HI) and waist-to-hip index (WHI) have been defined as the allometric equivalents of hip circumference and WHR, correspondingly, and are uncorrelated with BMI^15,16^.

In this study, we have examined, separately in women and men, whether a MR analysis provides evidence for a potentially causal association of genetically predicted morning plasma cortisol with body shape (as reflected in the allometric indices WHI, ABSI, and HI) and with general obesity (as reflected in BMI). To highlight the similarity of traditional body shape indices with BMI and their difference from allometric body shape indices, we have compared WHR with BMI and WHI.

## Methods

### Overview

MR analysis involves the use of genetic variants in an IV analysis framework and is based on the principle that genotypes are not associated with environmental confounders and are not affected by reverse causation^8^. The following three assumptions must be satisfied for the genetic instrument and the corresponding causal inference to be deemed valid: (i) the genetic variants are strongly associated with the exposure (morning plasma cortisol); (ii) the genetic variants have no direct effect on the outcome (body shape and obesity) and influence the outcome only via the exposure (morning plasma cortisol); (iii) the genetic variants are not associated with confounders (measured or unmeasured) of the exposure-outcome association.

We conducted a two-sample MR study, using publicly available summary statistics for gene-exposure and gene-outcome association estimates based on distinct and non-overlapping populations consisting of participants with European ancestry. We report our findings according to the MR-STROBE guidance^17^.

### Data sources

The genetic instrument for the exposure, morning plasma cortisol, was based on publicly available summary statistics from the latest GWAMA update of the CORNET consortium, which included 8,452,427 SNPs for up to 25,314 individuals from 17 population-based cohorts of European ancestry^10^. Linear regression had been performed jointly in women and men with z-scores of log-transformed morning plasma cortisol (standard deviation (SD) scale) and adjustment for sex, age, and ten cohort-specific principal components of genetic ancestry^10^. Sex-specific analyses were not available, as the authors had explained in their previous GWAMA, that they could not identify sex differences when examining separately women and men^9^.

Sex-specific genetic variants associated with the outcome body shape were extracted from the publicly available summary statistics of a genome-wide association study (GWAS) of allometric body shape indices based on 219,872 women and 186,825 men of European ancestry from the UK Biobank cohort^15^. WHI, ABSI, and HI had been calculated according to the general formula: Z × Weight^β^ × Height^γ^, where Z represented either WHR, waist circumference, or hip circumference, correspondingly, and the power coefficients β and γ had been calibrated for UK Biobank, i.e. they were derived based on UK Biobank data. Bayesian linear mixed-models (BOLT-LMM) had been performed, following inverse normal transformation of WHI, ABSI, and HI to SD scale (normalised SD-unit) using Blom’s method, and adjusting the models for age, age^2^, and an indicator of genotyping array^15^. Analyses combining women and men were not available.

Sex-specific genetic variants associated with the outcome general obesity reflected in BMI and with the traditional body shape index WHR were extracted from the publicly available summary statistics of a meta-analysis based on individuals of European ancestry (434,794 women and 374,756 men for BMI; 381,152 women and 316,772 men for WHR)^18^, which combined a GWAS with a linear mixed model in UK Biobank data and publicly available GWAS summary statistics obtained from the Genetic Investigation of Anthropometric Traits (GIANT) consortium^19^. BMI and WHR were expressed in SD units. No GWAS summary statistics of equivalent quality were available for waist and hip circumferences.

### Selection of genetic instruments

To improve the reliability of our findings, we included in the selection of genetic instruments SNPs with gene-exposure associations estimated in at least 20,000 individuals (7,300,058 SNPs, 86.4% of all available SNPs). We used the SNP2GENE function of the web-based platform for Functional Mapping and Annotation (FUMA) v1.6.0 to perform positional mapping, clumping, and annotation of genetic variants^20^. Linkage disequilibrium (LD) mapping was based on the *1000G Phase3 EUR* reference panel. All relevant genetic variants were mapped to genes within a maximum distance of 1 kb based on their genomic position on GRCh37 (hg19) and Ensemble genes v110 with ANNOVAR employed in FUMA^21^. We used the deleteriousness score (Combined Annotation Dependent Depletion (CADD) score) provided by FUMA as a measure of pathogenicity of a given genetic variant and evaluated this with respect to the recommended cut-off 12.37^22^.

In our main analysis, following the algorithm and terminology used in FUMA^20^, independent significant SNPs were defined as genetic variants associated with the exposure with significance p < 5 × 10^–6^ and only in a weak LD with each other at r^2^ < 0.6 (first clumping step). Genetic variants associated with the exposure at p < 0.05 and in LD with an independent significant SNP at r^2^ ≥ 0.6 constituted the corresponding high-LD block with candidate SNPs. In a second clumping step, independent significant SNPs were clumped to identify lead SNPs associated with the exposure at p < 5 × 10^–6^ and independent from each other at r^2^ < 0.05 (the lowest r^2^ accepted by FUMA). The lead SNPs identified by FUMA constituted the main genetic instrument (IV_A_). Lead SNPs with LD block boundaries closer than 250 kb were joined in a genetic locus. We used a more lenient significance criterion in our main analysis (p < 5 × 10^–6^) to allow for the inclusion of a wider range of genetic loci, since the conventional conservative genome-wide significance level (p < 5 × 10^–8^) had previously identified only the CBG-related locus on chromosome 14^10^. We applied, however, a conservative LD threshold (r^2^ < 0.05 rather than r^2^ < 0.3 used in Ref.^10^), to ensure independence of the selected SNPs. We used FUMA for IV selection because the lead SNPs are equivalent to clumping with PLINK at the specified p-value and r^2^ thresholds, but FUMA additionally permits a visualisation of the LD structure of the relevant genetic region^20^.

In a secondary analysis, we used FUMA to obtain an alternative genetic instrument (IV_B_), defining lead SNPs with a conservative genome-wide significance threshold at p < 5 × 10^–8^ but with a lenient threshold for independence at r^2^ < 0.3, similarly to Crawford et al.^10^.

We orientated the genetic variants of the outcome such that the effect allele corresponded to the minor allele (mean allele frequency ≤ 0.5). We aligned the genetic variants of the exposure to match the minor allele of the outcome, irrespective of the allele frequency of the exposure. Thus, the signs of the gene-outcome and gene-exposure association estimates for each genetic variant correspond to the allele representing the minor allele in the morning plasma cortisol dataset. For lead SNPs with unavailable gene-exposure association estimates, we used replacement candidate SNPs with p < 5 × 10^–6^ and r^2^ ≥ 0.6 with the corresponding lead SNP, when such were available. In the text below, SNPs are labelled with *chromosome_rsID*.

To characterise further the genetic determinants of morning plasma cortisol, we performed gene-based association analysis with Mutimarker Analysis of GenoMic Annotation (MAGMA) v.1.08^23^, including only genetic variants with association estimates based on at least 20,000 individuals. Gene-based analysis derives a SNP-wide mean model for each individual protein-coding gene. Significant were considered genes with p < 0.05, after Bonferroni correction for the number of identified protein-coding genes. Due to the limited power of the available GWAMA of morning plasma cortisol, we used gene Q-Q plots to identify additional genes of potential interest which showed substantial nominal significance but could not reach the Bonferroni adjusted significance cut-off.

### Statistical MR analysis

To calculate causal estimates for each individual SNP included in the genetic instrument, we used the Wald ratio (SNP-outcome divided by SNP-exposure regression coefficient)^24^. To provide some information for potential pathogenicity, we performed sensitivity analyses with the corresponding high-CADD analogue, defined as the candidate SNP from the corresponding LD block with p < 5 × 10^–5^ and the highest CADD > 12.37, when SNPs fulfilling these criteria were available.

To calculate causal estimates for morning plasma cortisol overall, we combined the SNP-specific Wald ratio estimates in an inverse variance weighted (IVW) analysis^24^. In our main analysis (IV_A_), which included independent SNPs across the genome, we used random-effects IVW because this allows the mean effects of the individual SNPs to differ due to horizontal pleiotropy and provides an unbiased estimate when horizontal pleiotropy is balanced^25^. In our secondary analysis (IV_B_), which included partly correlated SNPs from the same genetic locus, we used fixed-effect IVW because this assumes that all SNPs have the same effect. In sensitivity analysis, we performed fixed-effect IVW for IV_A_ and random-effects IVW for IV_B_, to check the robustness of our findings. For validation, we examined heterogeneity in the IVW estimates with the Cochran *Q* statistic and the corresponding test for heterogeneity (p_heterogeneity_) and the I^2^ metric of inconsistency^25^. To detect outliers, we additionally performed a sensitivity analysis with MR-PRESSO (Mendelian Randomization Pleiotropy RESidual Sum and Outlier), which includes a global test for heterogeneity (considering all SNPs jointly), a local test for heterogeneity (identifying outlier SNPs), and a distortion test (comparing the causal estimate before and after removal of the detected outliers)^26^. To assess horizontal pleiotropy, we used as sensitivity analyses two methods which make different IV assumptions: (i) weighted median method, which allows for some SNPs to be invalid instruments, as long as they account for less than 50% of the information^27^; (ii) MR-Egger regression, which provides consistent estimates when all SNPs are invalid, conditional on the InSIDE (Instrument Strength Independent of Direct Effect) assumption, stating that the direct (pleiotropic) effects of the genetic variants on the outcome are independent of the associations of the genetic variants with the exposure^28^. We used the MR-Egger intercept to evaluate potential IV violations, as this provides an estimate of the average pleiotropic effect and a test for directional pleiotropy (an intercept with p-value < 0.05 indicates the presence of horizontal pleiotropy)^28^.

To assess the strength of each SNP as a genetic instrument, we calculated an F statistic using as approximation the squared ratio of the gene-exposure association and the corresponding standard error (β_X_^2^/σ_X_^2^). To minimise weak instrument bias, we considered as acceptable strength of the gene-exposure association F > 10^29^. To evaluate the reliability of MR Egger estimates, we used the I^2^_GX_ statistic, which when low (< 90%) indicates a violation of the NOME (NO Measurement Error) assumption that the exposure is measured without measurement error^30^.

The estimates obtained from the MR analysis quantify the change in each outcome on an SD scale (SD_change_) per one SD increase in genetically predicted morning plasma cortisol. All p-values were two-sided. MR associations with p < 0.05 were considered statistically significant.

Data analyses and visualisation were performed with R version 4.1.3 (using the “MendelianRandomisation” v.0.7.0 and “ggplot2” v.3.3.5 packages)^31^. MR-PRESSO was run in R version 4.3.1.

### Ethics approval and consent to participate

This research did not involve individual level data and used only publicly available summary statistics generated from previously published studies, referenced in the manuscript, which had obtained ethics approval and informed consent from study participants in accordance with the Declaration of Helsinki.

## Results

### Genetic instruments

**Figure 1:**
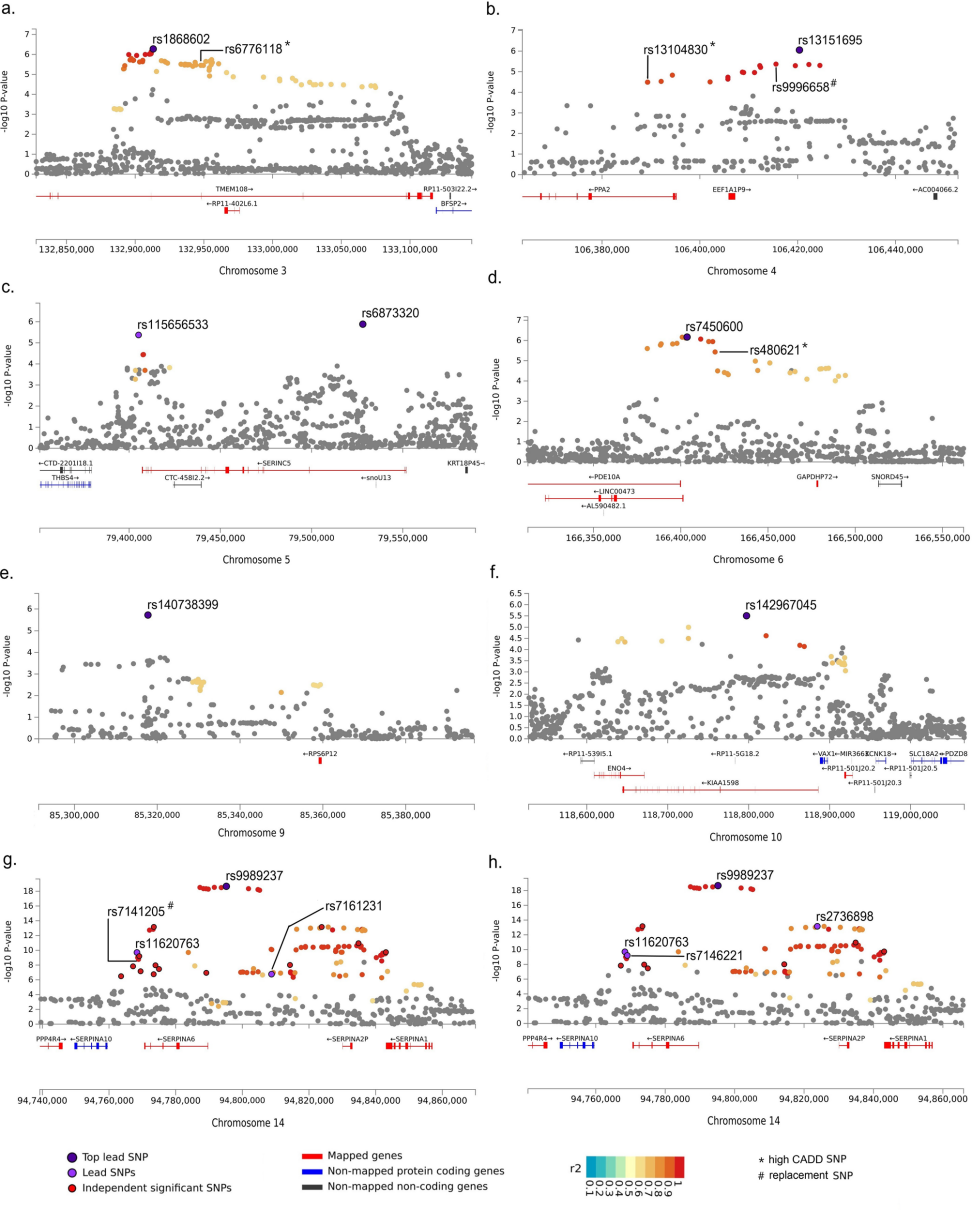
Locus plots for genetic variants associated with morning plasma cortisol. *CADD* Combined Annotation Dependent Depletion (CADD) score (index of pathogenicity), *IV*_*A*_ main instrumental variables set, derived with FUMA (Functional Mapping and Annotation) (p < 5** × **10^–6^ for the gene-exposure association, linkage disequilibrium r^2^ < 0.05), *IV*_*B*_ secondary instrumental variables set, derived with FUMA (p < 5 × 10^−8^, r^2^ < 0.3), same as in Crawford et al.^10^, *SNP* single nucleotide polymorphism. (**a**) Chromosome 3 for IV_A_ (n = 75 candidate SNPs in the LD block of the locus); (**b**) Chromosome 4 for IV_A_ (n = 17); (**c**) Chromosome 5 for IV_A_ (n = 8); (**d**) Chromosome 6 for IV_A_ (n = 30); (**e**) Chromosome 9 for IV_A_ (n = 20); (**f**) Chromosome 10 for IV_A_ (n = 21); (**g**) Chromosome 14 for IV_A_ (n = 114); (**h**) Chromosome 14 for IV_B_ (n = 101).

Our main selection strategy (p < 5 × 10^–6^, r^2^ < 0.05) identified a genetic instrument including ten lead SNPs (IV_A_): three in the *SERPINA6* locus (*14_rs11620763, 14_rs9989237, 14_rs7161231*) and seven on other chromosomes: *3_rs1868602 (*mapped to *TMEM108* gene on chromosome 3*), 4_rs13151695 (EEF1A1P9), 5_rs115656533 (SERINC5)*, 5_*rs6873320 (SERINC5), 6_rs7450600 (LINC00473), 9_rs140738399 (RPS6P12), 10_rs142967045 (KIAA1598)* (Table 1). Morning plasma cortisol was lower for the minor allele of *4_rs13151695, 5_rs115656533*, 5_*rs6873320, 6_rs7450600, 10_rs142967045,* and *14_rs11620763* and higher for the minor allele of the other four lead SNPs*.* Summary statistics for all ten lead SNPs were available for allometric body shape indices. Three SNPs, however, were unavailable for BMI and WHR, so we replaced *4_rs13151695* with *4_rs9996658* (r^2^ = 1.000 between the two SNPs) and *14_rs11620763* with *14_rs7141205* (r^2^ = 1.000), but there was no appropriate replacement for *9_rs140738399*. Thus, the adapted IV_A_^#^ included nine lead SNPs in the analyses for BMI and WHR. To facilitate the comparisons with WHR, we performed an additional matching analysis for WHI, using the adapted IV_A_^#^. None of the SNPs included in IV_A_ or their substitutes had substantial deleteriousness score (largest CADD = 7.24). There were, however, high-LD candidate SNPs with CADD > 12.37 for three of the lead SNPs. Thus, *3_rs6776118* was the high-CADD analogue for *3_rs1868602* (r^2^ = 0.748 between the two SNPs), *4_ rs13104830* for *4_rs13151695* (r^2^ = 0.946), and *6_rs480621* for *6_rs7450600* (r^2^ = 0.901) (Table 1, Fig. 1).

**Table 1:** Genetic instruments for the exposure, morning plasma cortisol.

rsID	CHR	Position	Gene	EA	NEA	EAF	MAF	Beta	SE	p-value	F	CADD	r^2^
IV_A_													
rs1868602	3	132,913,122	TMEM108	T	C	0.274	0.289	0.0567	0.0113	5.29 × 10^–7^	25.2	2.14	–
rs13151695^#^	4	106,420,356	EEF1A1P9	C	T	0.290	0.275	− 0.0569	0.0116	8.87 × 10^–7^	24.1	2.91	–
rs115656533	5	79,405,179	SERINC5	T	A	0.164	0.166	− 0.0693	0.0151	4.24 × 10^–6^	21.1	0.79	–
rs6873320	5	79,528,084	SERINC5	A	G	0.353	0.393	− 0.0551	0.0114	1.31 × 10^–6^	23.4	0.85	–
rs7450600	6	166,403,646	LINC00473	C	T	0.103	0.085	− 0.0837	0.0168	6.76 × 10^–7^	24.8	2.92	–
rs140738399^#^	9	85,317,761	RPS6P12	G	A	0.053	0.066	0.1182	0.0248	1.91 × 10^–6^	22.7	1.71	–
rs142967045	10	118,797,350	KIAA1598	T	C	0.030	0.022	− 0.1202	0.0258	3.12 × 10^–6^	21.7	0.77	–
rs11620763^#^	14	94,768,392	SERPINA6	A	G	0.193	0.197	− 0.0858	0.0135	1.97 × 10^–10^	40.4	0.34	–
rs9989237^£^	14	94,795,202	SERPINA6	T	C	0.210	0.205	0.0857	0.0095	2.16 × 10^–19^	81.4	0.57	–
rs7161231	14	94,808,760	SERPINA6	T	C	0.101	0.105	0.0677	0.0129	1.71 × 10^–7^	27.5	5.86	–
IV_replacements_^#^													
rs9996658	4	106,415,560	EEF1A1P9	A	C	0.292	0.275	− 0.0511	0.0111	4.29 × 10^–6^	21.2	7.24	1.000
rs7141205^£^	14	94,768,859	SERPINA6	G	A	0.195	0.197	− 0.0612	0.0102	1.68 × 10^–9^	36.0	1.82	1.000
IV_high-CADD_													
rs6776118	3	132,947,776	TMEM108	T	A	0.260	0.259	0.0524	0.0113	3.52 × 10^–6^	21.5	12.42	0.748
rs13104830	4	106,389,297	PPA2	T	C	0.296	0.286	− 0.0456	0.0110	3.23 × 10^–5^	17.2	12.91	0.946
rs480621	6	166,419,693	LINC00473	T	G	0.101	0.084	− 0.0781	0.0169	3.69 × 10^–6^	21.4	19.53	0.901
IV_B_													
rs11620763^#^	14	94,768,392	SERPINA6	A	G	0.193	0.197	− 0.0858	0.0135	1.97 × 10^–10^	40.4	0.34	–
rs7146221	14	94,769,081	SERPINA6	A	G	0.454	0.456	− 0.0504	0.0082	6.28 × 10^–10^	37.8	1.23	–
rs9989237^£^	14	94,795,202	SERPINA6	T	C	0.210	0.205	0.0857	0.0095	2.16 × 10^–19^	81.4	0.57	–
rs2736898^£^	14	94,823,817	SERPINA2P	T	C	0.497	0.491	0.0585	0.0078	7.03 × 10^–14^	56.3	4.00	–

*Beta* effect (regression coefficient), *CHR* chromosome, *CADD* Combined Annotation Dependent Depletion (CADD) score (index of pathogenicity), *EA* effect allele (the minor allele with mean allele frequency ≤ 0.5), *EAF* mean frequency of the minor allele from the genome-wide association meta-analysis (GWAMA) in Crawford et al*.*^10^, *F* F statistic, *MAF* minor allele frequency based on the reference panel (*1000G Phase3 EUR*) in FUMA (Functional Mapping and Annotation), *NEA* non-effect allele, *r*^*2*^ linkage disequilibrium r^2^ with the corresponding genetic variant in IV_A_, *SE* standard error, *IV*_*A*_ main instrumental variables set, derived with FUMA (p < 5 × 10^–6^ for the gene-exposure association, linkage disequilibrium r^2^ < 0.05), *IV*_*B*_ secondary instrumental variables set, derived with FUMA (p < 5 × 10^–8^, r^2^ < 0.3), same as in Crawford et al.^10^, *IV*_*replacements*_^*#*^ replacements for missing genetic variants used in the analyses for body mass index and waist-to-hip ratio (*9_rs140738399* was omitted due to lack of suitable replacement), *IV*_*high-CADD*_ genic variants with CADD > 12.37 in high LD (r^2^ ≥ 0.6) with the corresponding variants from the main IV_A_ set.

^£^Significant heterogeneity between the studies included in the GWAMA^10^: p = 0.0007 for *rs9989237*; p = 0.045 for *rs7141205*; p = 0.026 for *rs2736898*.

The secondary selection strategy (p < 5 × 10^–8^, r^2^ < 0.3) reproduced as lead SNPs for IV_B_ the four SNPs identified by Crawford et al.^10^ in the *SERPINA6* locus (*14_rs11620763, 14_rs7146221, 14_rs9989237, 14_rs2736898*) (Table 1, Fig. 1).

The F statistics for SNPs outside chromosome 14 were around 20 (17.2 to 25.2) and were higher for SNPs within the *SERPINA6* locus (27.5 to 81.4), but only SNPs on chromosome 14 had shown significant heterogeneity between the studies contributing to the GWAMA (Table 1).

Few lead SNPs showed gene-outcome associations with nominal statistical significance, except *6_rs7450600* and its high-CADD analogue *6_rs480621 (LINC00473)*, which were associated positively with HI and BMI in women and men (Supplementary Table S1 for WHI, ABSI, and HI; Supplementary Table S2 for BMI).

In the gene-based analysis, only *SERPINA1* and *SERPINA6* were associated with morning plasma cortisol at significance level p < 0.05, after Bonferroni adjustment for 18,814 identified protein-coding genes. The gene Q–Q plot, however, indicated that five more genes had higher than expected nominal significance including (in descending order of the unadjusted p-value): *SERPINA10* (chromosome 14)*, ETNK1* (chromosome 12)*, TMEM108* (chromosome 3)*, SERINC5* (chromosome 5)*, and PPP4R4* (chromosome 14), all with p ≤ 0.0001 (Supplementary Fig. S1).

### MR associations

Only in women, morning plasma cortisol proxied genetically by IV_A_ was associated positively with WHI (SD_change_ = 0.035; 95% confidence interval (CI) 0.002–0.067; p = 0.036) and ABSI (SD_change_ = 0.039; 95% confidence interval (CI) 0.006–0.071; p = 0.021), with no evidence for heterogeneity between individual SNPs. There was no evidence for associations with WHI or ABSI in men, or with HI and BMI in women or men. There was, however, evidence for heterogeneity for HI in women and men and for WHI and BMI in men.

**Figure 2:**
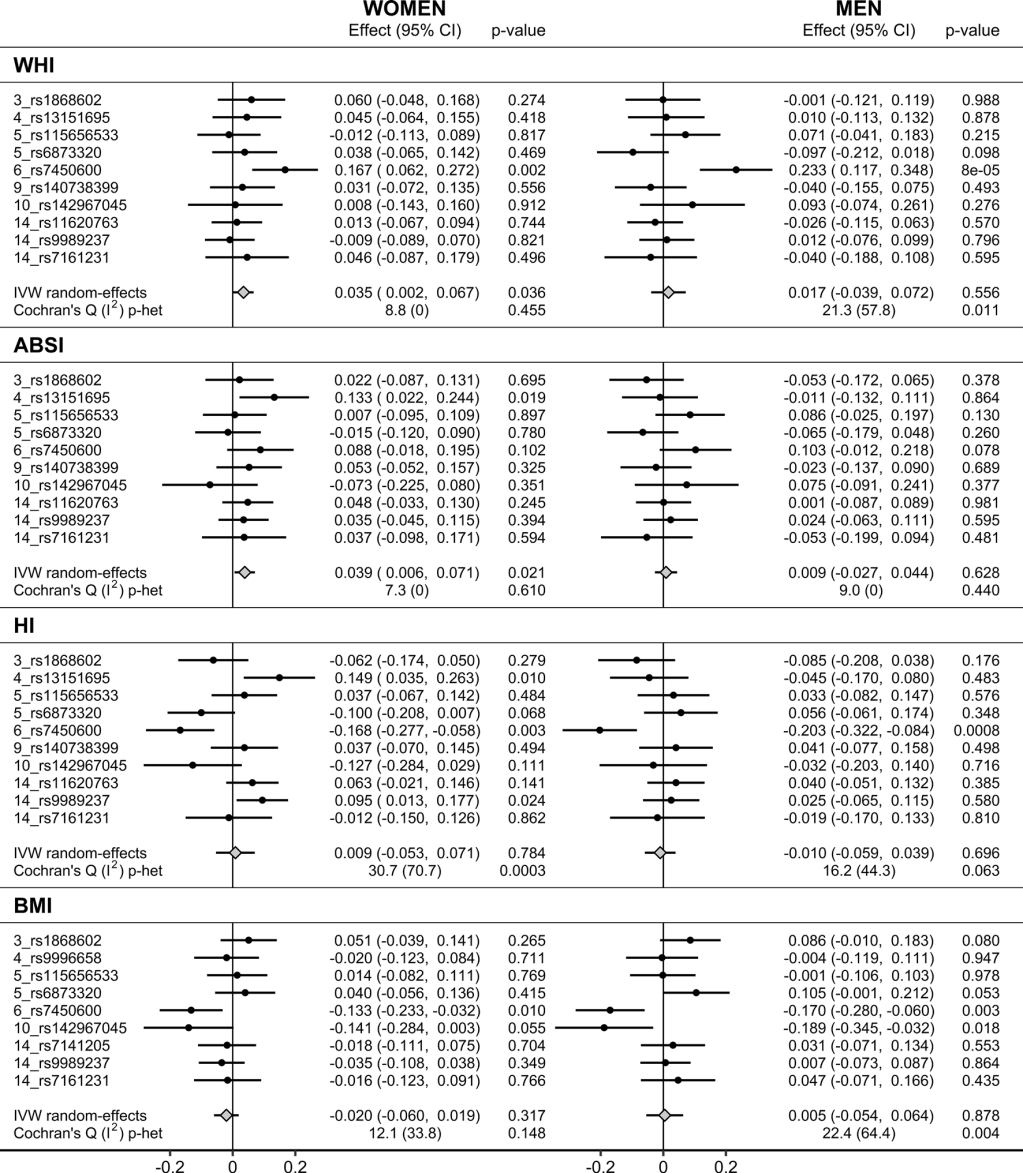
Associations of morning plasma cortisol genetically proxied by IV_A_. *ABSI* a body shape index, *BMI* body mass index, *CI* confidence interval, *Effect* Wald ratio (for individual SNPs) or IVW random-effects estimate, *HI* hip index, *IV*_*A*_ main instrumental variables set, derived with FUMA (Functional Mapping and Annotation) (p < 5 × 10^−6^ for the gene-exposure association, linkage disequilibrium r^2^ < 0.05), replacing for BMI *4_rs13151695* with *4_rs9996658* (r^2^ = 1.000), *14_rs11620763* with *14_rs7141205* (r^2^ = 1.000)*,* but omitting *9_rs140738399*, due to lack of suitable replacement; *IVW* inverse variance weighted analysis, *WHI* waist-to-hip index.

Among individual SNPs, morning plasma cortisol proxied genetically by *6_rs7450600* (*LINC00473*) was associated positively with WHI in both women (SD_change_ = 0.167; 95% CI 0.062–0.272; p = 0.002) and men (SD_change_ = 0.233; 95% CI 0.117–0.348; p = 8 × 10^–5^) (Fig. 1). There were associations with ABSI in the positive direction without nominal significance, but the positive associations with WHI were mainly accounted for by inverse associations with HI in both women (SD_change_ =  − 0.168; 95% CI − 0.277 to − 0.058; p = 0.003) and men (SD_change_ =  − 0.203; 95% CI − 0.322 to − 0.084; p = 0.0008). In addition, morning plasma cortisol proxied genetically by *6_rs7450600* was associated inversely with BMI in both women (SD_change_ =  − 0.133; 95% CI − 0.233 to − 0.032; p = 0.010) and men (SD_change_ =  − 0.170; 95% CI − 0.280 to − 0.060; p = 0.003). Only in women, morning plasma cortisol proxied genetically by *4_rs13151695* (*EEF1A1P9*) was associated positively with ABSI (SD_change_ = 0.133; 95% CI 0.022–0.244; p = 0.019) as well as with HI (SD_change_ = 0.149; 95% CI 0.035–0.263; p = 0.010) but not with WHI or BMI (Fig. 1). Further, in women and men, morning plasma cortisol proxied genetically by *10_rs142967045* (*KIAA1598*) was associated inversely with BMI, less prominently in women (SD_change_ =  − 0.141; 95% CI − 0.284 to 0.003; p = 0.055) than in men (SD_change_ =  − 0.189; 95% CI − 0.345 to − 0.032; p = 0.018). Last, only in women, morning plasma cortisol proxied genetically by *14_rs9989237* (*SERPINA6*) was associated positively with HI (SD_change_ = 0.095; 95% CI 0.013–0.177; p = 0.024) (Fig. 2).

**Figure 3:**
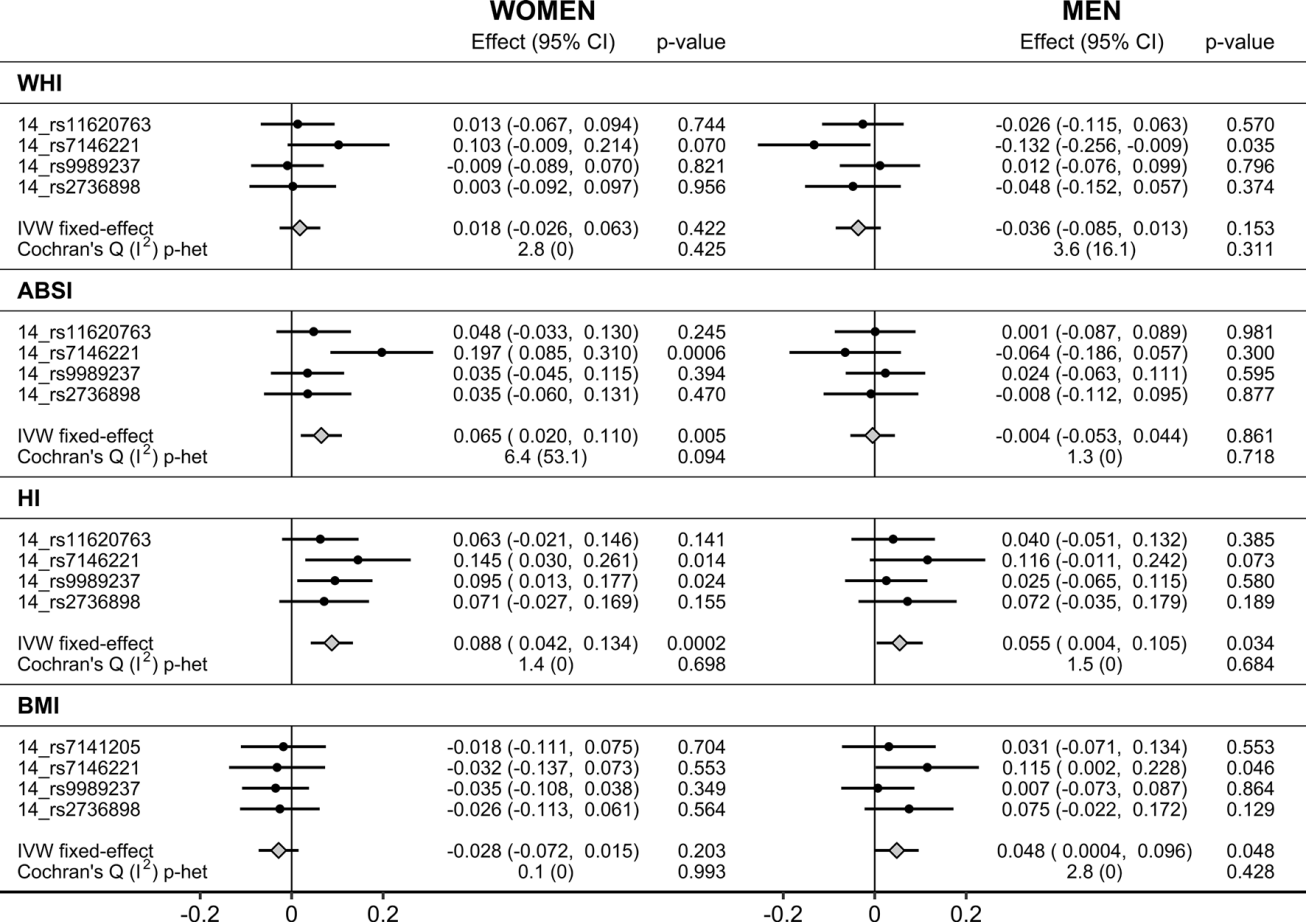
Associations of morning plasma cortisol genetically proxied by IV_B_. *ABSI* a body shape index, *BMI* body mass index, *CI* confidence interval, *Effect* Wald ratio (for individual SNPs) or IVW fixed-effect estimate, *HI* hip index, *IV*_*B*_ secondary instrumental variables set, derived with FUMA (Functional Mapping and Annotation) (p < 5 × 10^–8^ for the gene-exposure association, linkage disequilibrium r^2^ < 0.3), same as in Crawford et al.^10^, replacing for BMI *14_rs11620763* with *14_rs7141205* (r^2^ = 1.000), *IVW* inverse variance weighted analysis, *WHI* waist-to-hip index.

Morning plasma cortisol proxied genetically by IV_B_ (p < 5 × 10^–8^, r^2^ < 0.3) was associated positively with ABSI in women only (SD_change_ = 0.065; 95% CI 0.020–0.110; p = 0.005) and with BMI in men only (SD_change_ = 0.048; 95% CI 0.0004–0.096; p = 0.048). A positive association with HI was observed in both women (SD_change_ = 0.088; 95% CI 0.042–0.134; p = 0.0002) and men (SD_change_ = 0.055; 95% CI 0.004–0.105; p = 0.034). There was no evidence for heterogeneity between individual SNPs, although proxying morning plasma cortisol by *14_rs7146221* (*SERPINA6*), showed the strongest associations (Fig. 3).

### Sensitivity analyses

**Figure 4:**
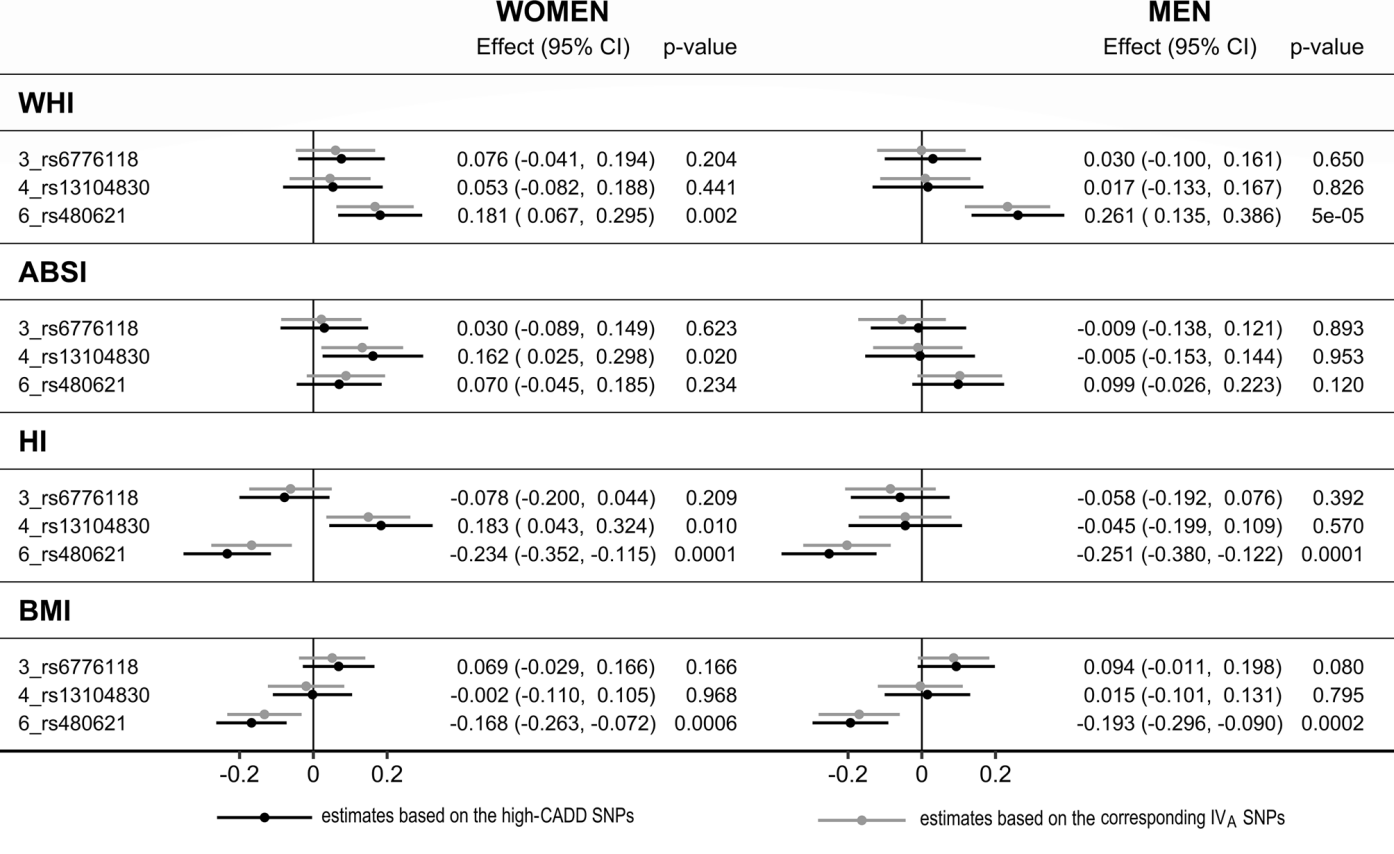
Associations of morning plasma cortisol genetically proxied by high-CADD variants. *ABSI* a body shape index, *BMI* body mass index, *CI* confidence interval, *Effect* Wald ratio estimate, *HI* hip index, *high-CADD* Combined Annotation Dependent Depletion (CADD) score (index of pathogenicity) above 12.37, *IV*_*A*_ the main instrumental variables set derived with FUMA (Functional Mapping and Annotation) (p < 5 × 10^−6^ for the gene-exposure association, linkage disequilibrium r^2^ < 0.05): the corresponding SNPs are *3_rs1868602* (r^2^ = 0.748)*, 4_rs13151695* (r^2^ = 0.946)*,* and *6_rs_7450600* (r^2^ = 0.901), *SNP* single nucleotide polymorphism, *WHI* waist-to-hip index.

Proxying morning plasma cortisol by the high-CADD variants *3_rs6776118*, *4_ rs13104830*, and *6_rs480621* showed similar association patterns to the corresponding lead SNPs in IV_A_ (*3_rs1868602*, *4_rs13151695*, and *6_rs7450600*) (Fig. 4).

**Figure 5:**
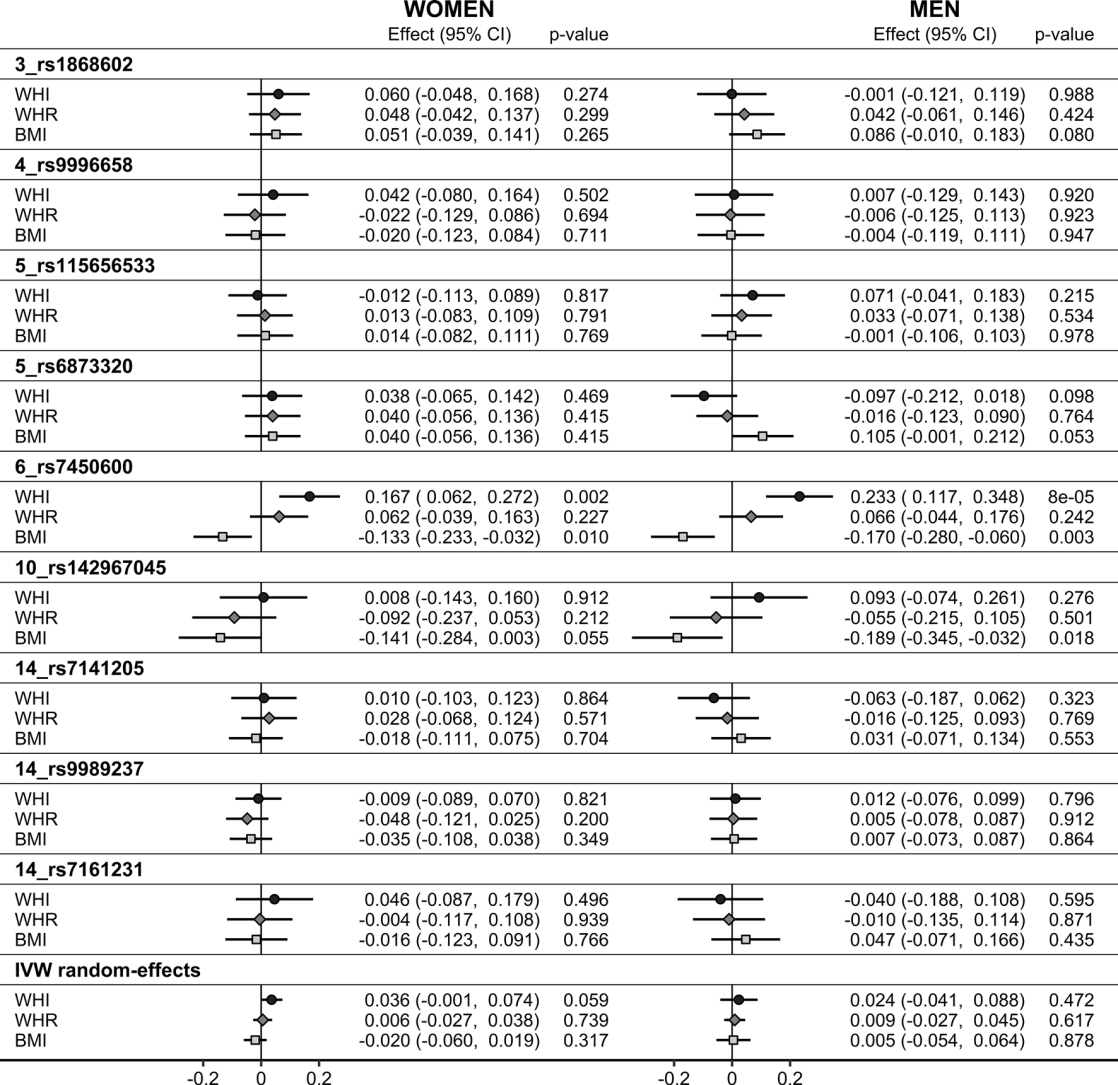
Associations of morning plasma cortisol genetically proxied by IV_A_^#^: comparisons between WHI, WHR, and BMI. *BMI* body mass index, *CI* confidence interval, *Effect* Wald ratio (for individual SNPs) or IVW random-effects estimate, *HI* hip index, *IV*_*A*_^*#*^ main instrumental variables set, derived with FUMA (Functional Mapping and Annotation) (p < 5 × 10^–6^ for the gene-exposure association, linkage disequilibrium r^2^ < 0.05), replacing *4_rs13151695* with *4_rs9996658* (r^2^ = 1.000), and *14_rs11620763* with *14_rs7141205* (r^2^ = 1.000)*,* but omitting *9_rs140738399*, due to lack of suitable replacement, *IVW* inverse variance weighted analysis, *WHI* waist-to-hip index, *WHR* waist-to-hip ratio.

Morning plasma cortisol proxied genetically by IV_A_^#^ showed intermediate associations with WHR compared to WHI and BMI, with the most prominent difference noted for *6_rs7450600*, for which a positive association with WHI and an inverse with BMI corresponded to a nil association with WHR in both women and men (Fig. 5).

Association estimates obtained with fixed-effect IVW and random-effects IVW were identical when there was no evidence for heterogeneity (low I^2^) and showed no material difference even when there was evidence for heterogeneity, except for a wider confidence interval of the positive association of morning plasma cortisol proxied by IV_B_ with ABSI in women when using random-effects IVW (SD_change_ = 0.065; 95% CI − 0.0006 to 0.131; p = 0.052) (Supplementary Table S3). The global test of MR-PRESSO, similarly to Cochran’s Q, indicated heterogeneity for HI in women and for WHI and BMI in men when using IV_A_, and the local tests identified *6_rs7450600* as an outlier, but there was no evidence for distortion and the conclusion of no association with these indices remained. Association estimates obtained with the weighted median method confirmed the findings of the main IVW random-effects analysis. There was no evidence for pleiotropy based on the MR Egger intercept, but MR Egger estimates had very wide confidence intervals for all analyses and low I^2^_GX_ < 50 (Supplementary Table S3).

## Discussion

Morning plasma cortisol was associated positively with WHI and ABSI in women, when proxied genetically by SNPs across the genome, and inversely with HI and BMI in women and men, when proxied individually by *6_rs7450600* in *LINC00473* locus. Morning plasma cortisol, however, was associated positively with HI in women and men when proxied genetically by SNPs confined to the CBG-related locus *SERPINA6*. Our findings are compatible with a causal association of higher morning plasma cortisol with larger waist size in women but show conflicting causal conclusions for hip size.

The interpretation of the associations of total morning plasma cortisol levels with any outcome should account for considerable biological and analytical caveats. Importantly, more than 90% of total blood cortisol is bound to CBG, while only free cortisol is traditionally considered biologically active^32^. On the one hand, CBG levels could be relevant to cortisol action since genetic variations in CBG can affect its affinity for cortisol and can modulate cortisol release and action at tissue level^33^. Crawford et al*.* have, indeed, shown that eQTL and GWAMA signals colocalise within a region of *SERPINA6* represented by *14_rs2736898* and have argued that this polymorphism can influence cortisol release at tissue level^10^. On the other hand, however, a similar functionality cannot be assumed for other polymorphisms in the *SERPINA6* locus and, when total plasma cortisol levels simply reflect CBG levels, the cortisol-driven negative feed-back to the hypothalamus-pituitary-adrenal (HPA) axis would adjust adrenal cortisol production and maintain constant free cortisol levels^34^. Further limiting the informativeness of circulating cortisol is the local interconversion between the active cortisol and the inactive cortisone by 11β-hydroxysteroid dehydrogenases, which regulate cortisol availability and action at tissue level^35^. Furthermore, a single cortisol measurement does not reflect the circadian and ultradian patterns of cortisol secretion and their alterations in disease states^36^. Similarly, blood levels are a short-term measure of cortisol status, while the glucocorticoid receptor (GR) shows a differential metabolic response to acute and chronic cortisol exposure, with lipolysis in the former and fat accumulation and obesity in the latter case^37^. There are also variations in the glucocorticoid sensitivity of the GR, which affect cortisol action at tissue level^38^. The above considerations determine the need to expand IVs for morning plasma cortisol outside the CBG-related *SERPINA6* region and to interpret with caution causal inference based solely on IVs in this region^39^. In addition to the biological considerations, there are also analytical limitations in cortisol measurements by immunoassays, which have been used by most studies contributing to the CORNET consortium^10^, as immunoassays have low specificity and can be subject to interference by cortisol precursors^40^. Such interferences would be particularly problematic in obesity, when there is an underlying adrenal disfunction but cortisol levels are not particularly high^5^. Bearing in mind the highlighted limitations of morning plasma cortisol measurements, we have compared below our findings with the results of previous studies and have discussed the MR assumptions.

The available knowledge on associations of cortisol with obesity is largely derived from population-based observational studies with a relatively small sample size and inconsistent findings, almost always with a cross-sectional design, often including only women or only men, and considering exclusively waist circumference and WHR as indices of body shape, with little attention paid to hip size^5^. Focusing on morning blood cortisol measurements, in agreement with our results, a meta-analysis of 26 observational studies found little evidence for associations with BMI, although there was a tendency towards lower levels in obese compared to non-obese individuals^41^. There was similarly little evidence for association with BMI on a continuous scale when using IVs located in *SERPINA6* in a two-sample MR^10^. A previously described inverse association with obesity class I (BMI ≥ 30 to < 35 kg/m^2^) and not above, although based on cortisol-related SNPs across the genome^12^, had derived IVs using the earlier smaller-size GWAMA and these did not overlap with our IVs. In contrast and in compliance with the expectations of the Cushing’s syndrome paradigm, a large meta-analysis of cortisol levels in hair, which are not dependent on CBG levels, provided robust observational evidence in support of a positive association with BMI (122 studies; 26,527 participants), including in the analyses restricted to studies using mass-spectrometry-based measurements as opposed to immunoassays^42^. Considering body shape indices, the associations with ABSI as a measure of waist size in our study were consistent between IV_A_, which included a wider range of genetic instruments, and IV_B_, which was confined to *SEPRINA6* locus. They also agree with the findings of the large meta-analysis of cortisol levels in hair, which reported positive associations with waist circumference (24 studies; 11,004 participants) and WHR (16 studies; 6,786 participants)^42^. For HI, however, which to date has only been examined in our study, there was a marked difference between the null association with IV_A_ and the positive association with IV_B_ in both women and men. Considering the relatedness of total plasma cortisol and CBG levels^32^, a positive association with HI likely reflects an influence of oestrogens on CBG levels, because CBG is higher in women compared to men^43^ and even higher in women using oestrogens^44^. At the same time, peripheral oestrogen production is highest in gluteofemoral adipose tissue^45^ and serum oestradiol is higher for larger HI^46^.

Notwithstanding the described associations, our study has shown that there is very little overlap between the genetic determinants of morning plasma cortisol, on the one hand, and body shape indices and BMI, on the other hand, especially given that the latter were based on biobank size datasets with thousands of SNPs reaching genome-wide significance^15,18^. This is not completely unexpected, since the genetic determinants of body shape indices reflected mainly links with morphogenesis and embryogenesis and not a dynamic influence of steroid hormones^15^. The main notable exception was *6_rs7450600* variant and its high-CADD analogue *6_rs480621* in *LINC00473* (Long Intergenic Non-Protein-Coding RNA 473) locus. These SNPs showed similar association patterns in women and men, which are thus more likely to be reproducible because sex-specific datasets could be considered a discovery and a validation cohort for each other. The *LINC00473* gene is especially interesting because the corresponding long non-coding RNA stimulates thermogenesis in brown adipose tissue and shows higher expression in supraclavicular than in abdominal subcutaneous adipose tissue but lower expression in obesity, which has impaired thermogenesis^47^. Given that in our study, BMI and HI were higher for the minor allele C of *6_rs7450600*, this would likely represent a loss of function variant. The corresponding lower morning plasma cortisol levels are compatible with the role of glucocorticoids for stimulation of thermogenesis^48^. LINC00473 is also considered oncogenic, as it is upregulated in various cancers^49^, promoting proliferation, migration, and invasion, and is associated with worse cancer survival^50^. Thus, the role of LINC00473 in cortisol-related pathology merits further investigation. Of some further interest for cortisol is also *TMEM108* (transmembrane protein 108) gene, which included multiple SNPs in LD and was ranked high in the gene-based MAGMA analysis. There was also a high-CADD analogue to the lead SNP but neither showed associations with body shape indices or BMI when used as genetic proxy of morning plasma cortisol in our study. *TMEM108* has not shown associations with body weight in animal studies either but has shown metabolic effects for glucose and lipid metabolism^51^.

Considering the MR assumptions, although our genetic variants were reasonably strongly associated with the exposure, a caveat remains that those outside the *SERPINA6* locus did not reach genome-wide significance and would require further validation. A major impediment is the low SNP-based heritability of morning plasma cortisol^52^, considerably lower than the estimates based on twin studies^53^. This drives a need for a substantial further increase in sample size of a future GWAMA and a need to examine genetic instruments for longer-term measures of cortisol exposure independent of CBG levels, such as cortisol levels in hair^52^. Regarding pleiotropy, it could be reasonably assumed that *SERPINA6* variants influence body shape only via modulating cortisol levels, but further knowledge of the mechanisms underlying the remaining variants in IV_A_ would be required to clarify their relationships with body shape, especially since some variants stood out with clearer and stronger associations than others. The MR Egger test for pleiotropy would not be reliable, given that a low I^2^_GX_ indicated that the NOME assumption is unlikely to hold. A violation of NOME is not unexpected, since cortisol exposure is determined by cortisol availability and action at cellular level, which may differ from total cortisol measured in blood due to the biological and analytical considerations discussed above. Regarding unmeasured confounders, these could not be disregarded either, as cortisol interacts biologically with sex steroids. Thus, in addition to increasing CBG levels^44^, oestrogens counter abdominal obesity via modification of the sensitivity of the glucocorticoid receptor^54^, while testosterone can supress adrenal cortisol production^55^ and is associated with body shape in a sex-specific pattern^46^. Multivariable analyses, however, were not feasible in our study because sex-steroid-related IVs suffer from the same limitation as cortisol-related IVs. Sex steroids are primarily bound to sex hormone binding globulin^56^ and their free fractions are unknown, their measurement is hampered by the low specificity and sensitivity of immunoassays and the diurnal and menstrual variations^57,58^, and they are subject to local tissue interconversions between the active and inactive forms^59^.

A strength of our study is the broader range of SNPs included in our genetic instrument, which was based on the latest and largest GWAMA of morning plasma cortisol. We have also used allometric indices to evaluate body shape independent of BMI and have re-iterated that traditional body shape indices resemble BMI in their associations and are thus unable to evaluate body shape independent of obesity. Nevertheless, a major limitation of our study is the relatively low power of the GWAMA of morning plasma cortisol compared to the biobank size GWAS of body shape indices and BMI, determining a need for validation of genetic variants outside the *SERPINA6* locus. Further, cortisol measurements in the studies contributing data to CORNET had been performed mainly with immunoassays^10^, which lack specificity and allow for a larger measurement error. Furthermore, while samples collected during the morning hours had been used, their timing and their relationship with the time of awakening had not been standardised, thus contributing to a larger variability. In addition, a single measurement would not reflect diurnal variations and longer-term cortisol status. Importantly, no sex-specific genetic association estimates for morning plasma cortisol were available, when HPA axis responsiveness shows sex differences^60^, and we were thus unable to derive sex-specific IVs. Similarly, age-specific estimates and separate estimates for pre- and post-menopausal women were also lacking, so we were unable to examine the influence of age and menopausal status on the observed associations. The publicly available summary statistics were derived from studies including only participants with European ancestry, so we could not examine ethnic differences. Not least, plasma cortisol as well as body shape are complex traits and are unlikely to fully reflect the underlying biological traits of interest, cortisol exposure at cellular level and body composition, correspondingly.

In conclusion, our findings support a causal association of higher morning plasma cortisol with larger waist size in women and highlight LINC00473 as a potential link between morning plasma cortisol levels and body shape, which merits further investigation.

### Supplementary Information

Supplementary Information.

## Data Availability

The datasets analysed in the current study are publicly available and can be accessed from the following locations. The summary statistics for morning plasma cortisol generated by Crawford et al.^10^, for women and men combined, can be downloaded from https://datashare.ed.ac.uk/handle/10283/3836. The summary statistics for allometric body shape indices generated by Christakoudi et al.^15^, separately for women and men, can be downloaded from the NHGRI-EBI GWAS Catalog at https://www.ebi.ac.uk/gwas/publications/34021172 (select option FTP Download). The summary statistics for BMI and WHR generated by Pulit et al.^18^, separately for women and men, can be downloaded from https://zenodo.org/record/1251813#.XCLJ7vZKhE4. The associated FUMA results will be made publicly available upon acceptance at https://fuma.ctglab.nl.

## Abbreviations

ABSI: A body shape index
BMI: Body mass index
CADD: Combined annotation dependent depletion score
CI: Confidence interval
CORNET: CORtisol NETwork consortium
FUMA: Functional Mapping and Annotation
GIANT: Genetic Investigation of Anthropometric Traits consortium
GWAMA: Genome-wide association meta-analysis
HI: Hip index
MAGMA: Multimarker Analysis of GenoMic Annotation
SD: Standard deviation
SNP: Single nucleotide polymorphism
WHI: Waist-to-hip index
WHR: Waist-to-hip ratio
