# Supplementary material for: Links between the genetic determinants of morning plasma cortisol and body shape: a two-sample Mendelian randomisation study
Source: Sci Rep. 2024 Feb 8;14:3230. doi: 10.1038/s41598-024-53727-4 (PMC10853188; doi:10.1038/s41598-024-53727-4)
Supplement: Supplementary file 1 — Supplementary Information. [file 41598_2024_53727_MOESM1_ESM.pdf]

Links between the genetic determinants of morning plasma cortisol and body shape: a two-sample Mendelian randomisation study

Sofia Christakoudi, Alexandros-Georgios Asimakopoulos, Elio Riboli, Konstantinos K. Tsilidis

Supplementary Tables

Supplementary Table S1 Summary statistics for the outcomes: WHI, ABSI, and HI 2

Supplementary Table S2 Summary statistics for the outcomes: WHI, BMI, WHR...................... 3

Supplementary Table S3 MR sensitivity analyses 4

Supplementary Figures

Supplementary Figure S1 Gene-based association analysis (MAGMA) 6

Supplementary Table S1 Summary statistics for the outcomes: WHI, ABSI, and HI

rsID	CHR	Position	INFO	EAF	B_{WHI}	SE_{WHI}	P_{WHI}	B_{ABSI}	SE_{ABSI}	P_{ABSI}	B_{HI}	SE_{HI}	P_{HI}
**WOMEN**													
**IV_A**													
rs1868602	3	132913122	0.979	0.291	0.0034	0.0031	0.27	0.0012	0.0032	0.69	-0.0035	0.0032	0.28
rs13151695	4	106420356	0.987	0.271	-0.0026	0.0032	0.42	-0.0076	0.0032	0.019	-0.0085	0.0033	0.010
rs115656533	5	79405179	0.950	0.203	0.0008	0.0036	0.82	-0.0005	0.0036	0.90	-0.0026	0.0037	0.48
rs6873320	5	79528084	0.967	0.396	-0.0021	0.0029	0.47	0.0008	0.0029	0.78	0.0055	0.0030	0.068
rs7450600	6	166403646	0.987	0.112	-0.0140	0.0045	0.002	-0.0074	0.0045	0.10	0.0140	0.0047	0.003
rs140738399	9	85317761	0.988	0.055	0.0037	0.0062	0.56	0.0062	0.0063	0.32	0.0044	0.0065	0.49
rs142967045	10	118797350	0.954	0.025	-0.0010	0.0093	0.91	0.0087	0.0094	0.35	0.0153	0.0096	0.11
rs11620763	14	94768392	0.957	0.212	-0.0012	0.0035	0.74	-0.0041	0.0036	0.25	-0.0054	0.0037	0.14
rs9989237	14	94795202	0.998	0.209	-0.0008	0.0035	0.82	0.0030	0.0035	0.39	0.0081	0.0036	0.024
rs7161231	14	94808760	0.989	0.107	0.0031	0.0046	0.50	0.0025	0.0046	0.59	-0.0008	0.0048	0.86
**$IV_{high\text{-}CADD}$**													
rs6776118	3	132947776	0.995	0.277	0.0040	0.0031	0.20	0.0016	0.0032	0.62	-0.0041	0.0033	0.21
rs13104830	4	106389297	1.000	0.278	-0.0024	0.0031	0.44	-0.0074	0.0032	0.020	-0.0084	0.0033	0.010
rs480621	6	166419693	0.988	0.109	-0.0142	0.0045	0.002	-0.0055	0.0046	0.23	0.0182	0.0047	0.0001
**IV_B**													
rs11620763	14	94768392	0.957	0.212	-0.0012	0.0035	0.74	-0.0041	0.0036	0.25	-0.0054	0.0037	0.14
rs7146221	14	94769081	0.976	0.460	-0.0052	0.0029	0.070	-0.0099	0.0029	0.0006	-0.0073	0.0030	0.014
rs9989237	14	94795202	0.998	0.209	-0.0008	0.0035	0.82	0.0030	0.0035	0.39	0.0081	0.0036	0.024
rs2736898	14	94823817	0.999	0.482	0.0002	0.0028	0.96	0.0021	0.0029	0.47	0.0042	0.0029	0.15
**MEN**													
**IV_A**													
rs1868602	3	132913122	0.979	0.291	-0.0001	0.0035	0.99	-0.0030	0.0034	0.38	-0.0048	0.0036	0.18
rs13151695	4	106420356	0.987	0.269	-0.0005	0.0035	0.88	0.0006	0.0035	0.86	0.0025	0.0036	0.48
rs115656533	5	79405179	0.950	0.203	-0.0049	0.0040	0.21	-0.0059	0.0039	0.13	-0.0023	0.0041	0.58
rs6873320	5	79528084	0.967	0.396	0.0053	0.0032	0.098	0.0036	0.0032	0.26	-0.0031	0.0033	0.35
rs7450600	6	166403646	0.987	0.113	-0.0195	0.0049	$8*10^{-5}$	-0.0086	0.0049	0.078	0.0170	0.0051	0.0008
rs140738399	9	85317761	0.988	0.055	-0.0047	0.0069	0.49	-0.0027	0.0068	0.69	0.0048	0.0071	0.50
rs142967045	10	118797350	0.954	0.025	-0.0112	0.0103	0.28	-0.0090	0.0102	0.38	0.0038	0.0105	0.72
rs11620763	14	94768392	0.957	0.212	0.0022	0.0039	0.57	-0.0001	0.0039	0.98	-0.0035	0.0040	0.39
rs9989237	14	94795202	0.998	0.210	0.0010	0.0038	0.80	0.0020	0.0038	0.59	0.0022	0.0039	0.58
rs7161231	14	94808760	0.989	0.106	-0.0027	0.0051	0.59	-0.0036	0.0051	0.48	-0.0013	0.0052	0.81
**$IV_{high\text{-}CADD}$**													
rs6776118	3	132947776	0.995	0.277	0.0016	0.0035	0.65	-0.0005	0.0035	0.89	-0.0031	0.0036	0.39
rs13104830	4	106389297	1.000	0.276	-0.0008	0.0035	0.83	0.0002	0.0035	0.95	0.0020	0.0036	0.57
rs480621	6	166419693	0.988	0.110	-0.0204	0.0050	$5*10^{-5}$	-0.0077	0.0050	0.12	0.0196	0.0051	0.0001
**IV_B**													
rs11620763	14	94768392	0.957	0.212	0.0022	0.0039	0.57	-0.0001	0.0039	0.98	-0.0035	0.0040	0.39
rs7146221	14	94769081	0.976	0.460	0.0067	0.0032	0.035	0.0032	0.0031	0.30	-0.0058	0.0032	0.073
rs9989237	14	94795202	0.998	0.210	0.0010	0.0038	0.80	0.0020	0.0038	0.59	0.0022	0.0039	0.58
rs2736898	14	94823817	0.999	0.482	-0.0028	0.0031	0.37	-0.0005	0.0031	0.88	0.0042	0.0032	0.19

ABSI – a body shape index; **B** – effect (regression coefficient); **CHR** – chromosome; **CADD** – Combined Annotation Dependent Depletion (CADD) score (index of deleteriousness); **EAF** – effect allele frequency in the body shape dataset; **HI** – hip index; **INFO** – imputation quality factor; **IV_A** – main instrumental variables set derived with FUMA (Functional Mapping and Annotation) ($p<5*10^{-6}$ for the gene-exposure association, linkage disequilibrium (LD) $r^2<0.05$); **IV_B** – secondary instrumental variables set, derived with FUMA ($p<5*10^{-8}$, $r^2<0.3$), same as in Crawford *et al*. [10]; **$IV_{high\text{-}CADD}$** – genic variants with CADD>12.37 in high LD ($r^2 \geq 0.6$) with the corresponding variants in the main IV_A set; **P** – p-value; **SE** – standard error; **WHI** – waist-to-hip index.

The sign of B and the value of EAF correspond to the allele with mean frequency ≤0.5 (minor allele) in the dataset for the exposure (morning plasma cortisol).

Supplementary Table S2 Summary statistics for the outcomes: WHI, BMI, WHR

rsID	CHR	Position	INFO	EAF	B_{WHI}	SE_{WHI}	P_{WHI}	B_{WHR}	SE_{WHR}	P_{WHR}	B_{BMI}	SE_{BMI}	P_{BMI}
**WOMEN**													
**IV_A #**													
rs1868602	3	132913122	0.979	0.285	0.0034	0.0031	0.27	0.0027	0.0026	0.31	0.0029	0.0026	0.26
rs9996658 #	4	106415560	0.992	0.271	-0.0021	0.0032	0.50	0.0011	0.0028	0.68	0.0010	0.0027	0.72
rs115656533	5	79405179	0.950	0.200	0.0008	0.0036	0.82	-0.0009	0.0034	0.80	-0.0010	0.0034	0.77
rs6873320	5	79528084	0.967	0.400	-0.0021	0.0029	0.47	-0.0022	0.0027	0.43	-0.0022	0.0027	0.41
rs7450600	6	166403646	0.987	0.107	-0.0140	0.0045	0.002	-0.0052	0.0043	0.23	0.0111	0.0043	0.009
rs142967045	10	118797350	0.954	0.024	-0.0010	0.0093	0.91	0.0111	0.0089	0.21	0.0169	0.0088	0.055
rs7141205 #	14	94768859	0.972	0.204	-0.0006	0.0035	0.86	-0.0017	0.0030	0.57	0.0011	0.0029	0.72
rs9989237	14	94795202	0.998	0.215	-0.0008	0.0035	0.82	-0.0041	0.0032	0.21	-0.0030	0.0032	0.34
rs7161231	14	94808760	0.989	0.109	0.0031	0.0046	0.50	-0.0003	0.0039	0.94	-0.0011	0.0037	0.77
**$IV_{high\text{-}CADD}$**													
rs6776118	3	132947776	0.995	0.273							0.0036	0.0026	0.17
rs13104830	4	106389297	1.000	0.287							0.0001	0.0025	0.95
rs480621	6	166419693	0.988	0.100							0.0131	0.0038	0.0005
**IV_B #**													
rs7141205 #	14	94768859	0.972	0.204							0.0011	0.0029	0.72
rs7146221	14	94769081	0.976	0.464							0.0016	0.0027	0.54
rs9989237	14	94795202	0.998	0.215							-0.0030	0.0032	0.34
rs2736898	14	94823817	0.999	0.493							-0.0015	0.0026	0.57
**MEN**													
**IV_A #**													
rs1868602	3	132913122	0.979	0.287	-0.0001	0.0035	0.99	0.0024	0.0030	0.41	0.0049	0.0028	0.081
rs9996658 #	4	106415560	0.992	0.272	-0.0004	0.0035	0.92	0.0003	0.0031	0.94	0.0002	0.0030	0.94
rs115656533	5	79405179	0.950	0.201	-0.0049	0.0040	0.21	-0.0023	0.0037	0.54	0.0001	0.0037	0.97
rs6873320	5	79528084	0.967	0.400	0.0053	0.0032	0.098	0.0009	0.0030	0.78	-0.0058	0.0030	0.049
rs7450600	6	166403646	0.987	0.107	-0.0195	0.0049	$8*10^{-5}$	-0.0055	0.0047	0.24	0.0142	0.0047	0.002
rs142967045	10	118797350	0.954	0.024	-0.0112	0.0103	0.28	0.0066	0.0098	0.50	0.0227	0.0096	0.019
rs7141205 #	14	94768859	0.972	0.204	0.0038	0.0039	0.32	0.0010	0.0034	0.78	-0.0019	0.0032	0.55
rs9989237	14	94795202	0.998	0.215	0.0010	0.0038	0.80	0.0004	0.0036	0.91	0.0006	0.0035	0.87
rs7161231	14	94808760	0.989	0.108	-0.0027	0.0051	0.59	-0.0007	0.0043	0.87	0.0032	0.0041	0.43
**$IV_{high\text{-}CADD}$**													
rs6776118	3	132947776	0.995	0.274							0.0049	0.0028	0.076
rs13104830	4	106389297	1.000	0.285							-0.0007	0.0027	0.80
rs480621	6	166419693	0.988	0.101							0.0151	0.0041	0.0003
**IV_B #**													
rs7141205 #	14	94768859	0.972	0.204							-0.0019	0.0032	0.55
rs7146221	14	94769081	0.976	0.464							-0.0058	0.0029	0.047
rs9989237	14	94795202	0.998	0.215							0.0006	0.0035	0.87
rs2736898	14	94823817	0.999	0.494							0.0044	0.0029	0.13

BMI – body mass index; **B** – effect (regression coefficient); **CHR** – chromosome; **CADD** – Combined Annotation Dependent Depletion (CADD) score (index of deleteriousness); **EAF** – effect allele frequency in the BMI dataset; **INFO** – imputation quality factor; **IV_A#** – main instrumental variables set derived with FUMA (Functional Mapping and Annotation) ($p<5*10^{-6}$ for the gene-exposure association, linkage disequilibrium (LD) $r^2<0.05$), replacing *4_rs13151695* with *4_rs9996658* ($r^2=1.000$), and *14_rs11620763* with *14_rs7141205* ($r^2=1.000$), but omitting *9_rs140738399*, due to lack of suitable replacement; **IV_B#** – secondary instrumental variables set, derived with FUMA ($p<5*10^{-8}$, $r^2<0.3$), same as in Crawford *et al*. [10], but replacing *14_rs11620763* with *14_rs7141205* ($r^2=1.000$); **$IV_{high\text{-}CADD}$** – genic variants with CADD>12.37 in high LD ($r^2\geq0.6$) with corresponding variants in the main IV_A set; **P** – p-value; **SE** – standard error; **WHI** – waist-to-hip index; **WHR** – waist-to-hip ratio.

The sign of B and the value of EAF correspond to the allele with mean frequency ≤0.5 (minor allele) in the dataset for the exposure (morning plasma cortisol).

Supplementary Table S3 MR sensitivity analyses

	WOMEN					MEN				
**Method**	**Effect (95% CI)**	**p**	**Q**	**p-het**	**I^2 / I^2_{GX}**	**Effect (95% CI)**	**p**	**Q**	**p-het**	**I^2 / I^2_{GX}**
**WHI – IV_A**										
IVW random-effects [$]	**0.035 (0.002, 0.067)**	**0.036**	8.8	0.455	0	0.017 (-0.039, 0.072)	0.556	21.3	**0.011**	57.8
IVW fixed-effect	-					0.017 (-0.019, 0.052)				
MR-PRESSO	-			0.462		-0.006 (-0.040, 0.028)	0.725		**0.018** [a]	
Weighted median	0.028 (-0.015, 0.071)	0.200				0.004 (-0.046, 0.053)	0.877			
MR Egger slope	0.009 (-0.135, 0.154)	0.898	8.7	0.371	32.2	0.104 (-0.138, 0.347)	0.399	20.0	**0.010**	32.8
MR Egger intercept	0.002 (-0.009, 0.012)	0.726				-0.007 (-0.024, 0.011)	0.465			
**WHI – IV_B**										
IVW random-effects	-					-0.036 (-0.090, 0.018)	0.190			
IVW fixed-effect [$]	0.018 (-0.026, 0.063)	0.422	2.8	0.425	0	-0.036 (-0.085, 0.013)	0.153	3.6	0.311	16.1
MR-PRESSO	-			0.476		-			0.383	
Weighted median	0.006 (-0.048, 0.059)	0.834				-0.029 (-0.090, 0.033)	0.364			
MR Egger slope	-0.101 (-0.297, 0.095)	0.313	1.3	0.524	21.4	0.146 (-0.071, 0.362)	0.188	0.7	0.693	21.4
MR Egger intercept	0.008 (-0.005, 0.022)	0.221				-0.013 (-0.028, 0.002)	0.092			
**ABSI – IV_A**										
IVW random-effects [$]	**0.039 (0.006, 0.071)**	**0.021**	7.3	0.610	0	0.009 (-0.027, 0.044)	0.628	9.0	0.440	0
IVW fixed-effect	-					-				
MR-PRESSO	-			0.679		-			0.461	
Weighted median	0.036 (-0.007, 0.079)	0.098				0.000 (-0.048, 0.048)	0.994			
MR Egger slope	0.025 (-0.115, 0.165)	0.723	7.2	0.513	32.3	0.109 (-0.044, 0.261)	0.162	7.2	0.513	32.8
MR Egger intercept	0.001 (-0.009, 0.011)	0.849				-0.007 (-0.019, 0.004)	0.186			
**ABSI – IV_B**										
IVW random-effects	0.065 (-0.0006, 0.131)	0.052				-				
IVW fixed-effect [$]	**0.065 (0.020, 0.110)**	**0.005**	6.4	0.094	53.1	-0.004 (-0.053, 0.044)	0.861	1.3	0.718	0
MR-PRESSO	-			0.194		-			0.740	
Weighted median	0.039 (-0.017, 0.095)	0.173				0.000 (-0.058, 0.058)	0.998			
MR Egger slope	-0.113 (-0.360, 0.134)	0.371	3.1	0.210	21.4	0.103 (-0.111, 0.318)	0.345	0.3	0.849	21.4
MR Egger intercept	0.013 (-0.004, 0.030)	0.148				-0.008 (-0.022, 0.007)	0.312			
**HI – IV_A**										
IVW random-effects [$]	0.009 (-0.053, 0.071)	0.784	30.7	**0.0003**	70.7	-0.010 (-0.059, 0.039)	0.696	16.2	0.063	44.3
IVW fixed-effect	0.009 (-0.025, 0.042)	0.612				-0.010 (-0.047, 0.027)	0.600			
MR-PRESSO	0.027 (-0.028, 0.082)	0.365		**<0.001** [b]		-			0.080	
Weighted median	0.037 (-0.015, 0.089)	0.158				0.028 (-0.022, 0.077)	0.276			
MR Egger slope	0.038 (-0.243, 0.319)	0.790	30.5	**0.0002**	32.3	0.020 (-0.202, 0.243)	0.857	16.0	**0.042**	32.8
MR Egger intercept	-0.002 (-0.023, 0.018)	0.832				-0.002 (-0.018, 0.014)	0.785			
**HI – IV_B**										
IVW random-effects	-					-				
IVW fixed-effect [$]	**0.088 (0.042, 0.134)**	**0.0002**	1.4	0.698	0	**0.055 (0.004, 0.105)**	**0.034**	1.5	0.684	0
MR-PRESSO	-			0.774		-			0.705	
Weighted median	**0.079 (0.023, 0.134)**	**0.006**				0.044 (-0.017, 0.106)	0.155			
MR Egger slope	0.017 (-0.186, 0.220)	0.872	0.9	0.627	21.4	-0.075 (-0.297, 0.147)	0.506	0.1	0.949	21.4
MR Egger intercept	0.005 (-0.009, 0.019)	0.480				0.009 (-0.006, 0.024)	0.239			
**BMI – IV_A**										
IVW random-effects [$]	-0.020 (-0.060, 0.019)	0.317	12.1	0.148	33.8	0.005 (-0.054, 0.064)	0.878	22.4	**0.004**	64.4
IVW fixed-effect	-0.020 (-0.052, 0.012)	0.219				0.005 (-0.031, 0.040)	0.797			
MR-PRESSO	-			0.173		0.024 (-0.024, 0.073)	0.351		**0.008** [c]	
Weighted median	-0.018 (-0.062, 0.026)	0.425				0.014 (-0.036, 0.064)	0.589			
MR Egger slope	**-0.220 (-0.378, -0.062)**	**0.006**	5.7	0.577	23.9	**-0.278 (-0.503, -0.053)**	**0.015**	11.8	0.108	24.4
MR Egger intercept	**0.013 (0.003, 0.024)**	**0.011**				**0.019 (0.004, 0.034)**	**0.012**			
**BMI – IV_B**										
IVW random-effects	-					-				
IVW fixed-effect [$]	-0.028 (-0.072, 0.015)	0.203	0.1	0.993	0	**0.048 (0.0004, 0.096)**	**0.048**	2.8	0.428	0
MR-PRESSO	-			0.991		-			0.466	
Weighted median	-0.030 (-0.080, 0.020)	0.242				0.037 (-0.023, 0.098)	0.225			
MR Egger slope	-0.044 (-0.270, 0.182)	0.701	0.1	0.967	21.6	-0.143 (-0.389, 0.104)	0.257	0.4	0.829	16.0
MR Egger intercept	0.001 (-0.013, 0.015)	0.888				0.012 (-0.003, 0.028)	0.121			

ABSI – a body shape index; **CI** – confidence interval; **HI** – hip index; **IVW** – inverse variance weighted analysis; **MR** – Mendelian randomisation; **MR-PRESSO** – Mendelian Randomization Pleiotropy RESidual Sum and Outlier; **WHI** – waist-to-hip index; **WHR** – waist-to-hip ratio.

IV_A – main instrumental variables set derived with FUMA (Functional Mapping and Annotation) ($p<5*10^{-6}$ for the gene-exposure association, linkage disequilibrium $r^2<0.05$).

IV_B – secondary instrumental variables set derived with FUMA ($p<5*10^{-8}$, $r^2<0.3$), same as in Crawford *et al*. [10].

[$] – main IVW model, shown in Figure 2 (IV_A) and Figure 3 (IV_B).

p – p-value of the effect estimate (p<0.05 in **bold**).

Q – Cochran's Q statistic.

p-het – p-value associated with Cochran's Q statistic (based on χ^2 distribution with n-1 degrees of freedom, n – number of single nucleotide polymorphisms, SNPs) or p-value for MR-PRESSO global test (based on a non-parametric simulation procedure) (p<0.05 in **bold**).

I^2 / I^2_{GX} – I^2 corresponds to the IVW analysis and I^2_{GX} corresponds to the MR Egger analysis.

Cochran's Q and I^2 were identical for fixed-effect IVW and random-effects IVW and the association estimates were also identical when there was no evidence for heterogeneity (low I^2).

Outliers (SNPs) detected with MR-PRESSO (p-value for distortion test – compares the causal estimate before and after removal of the detected outliers):

[a] – rs7450600 (p=0.672)

[b] – rs7450600 (p=0.107)

[c] – rs7450600 (p=0.627)

MR-PRESSO did not report association estimates when the global test was not statistically significant.

a.

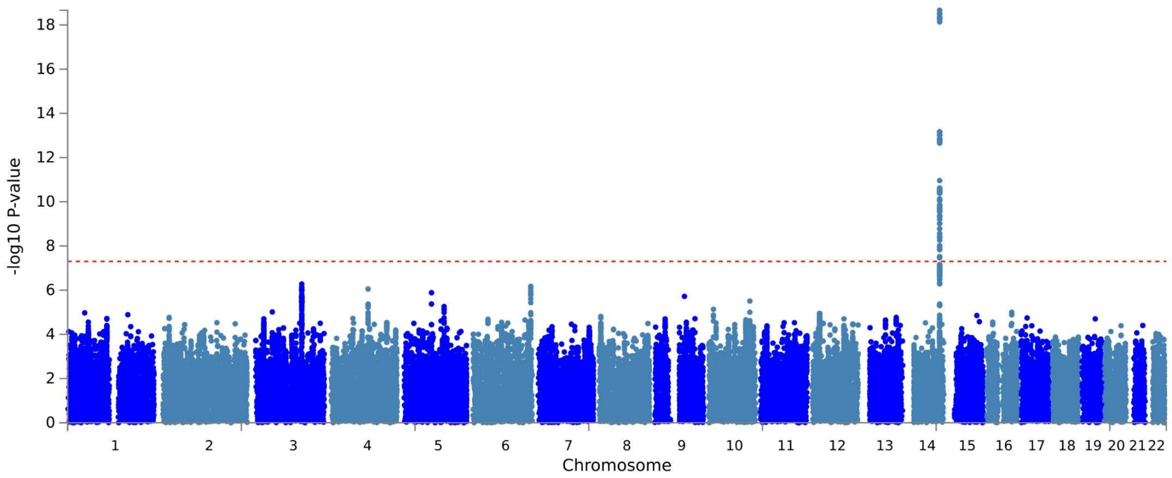

b.

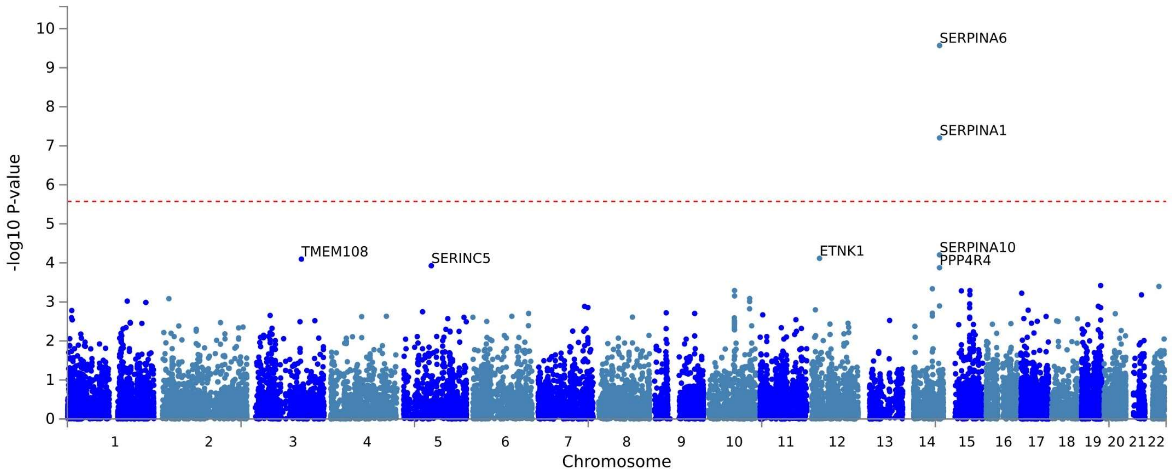

c.

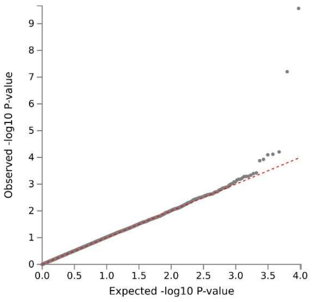

d.

CHR	SYMBOL	START	STOP	N SNPS	N PARAM	N	ZSTAT	P	P_{ADJ}
3	TMEM108	132756235	133117636	1069	42	22687	3.77	$8.0*10^{-5}$	1
5	SERINC5	79406050	79552898	469	45	22201	3.68	0.00011	1
12	ETNK1	22777009	22844599	101	10	24426	3.79	$7.7*10^{-5}$	1
14	PPP4R4	94611465	94747072	301	17	23811	3.65	0.00013	1
14	SERPINA10	94748650	94760608	32	7	24004	3.84	$6.2*10^{-5}$	1
14	SERPINA6	94769585	94790731	89	14	24673	6.21	$2.7*10^{-10}$	$5.1*10^{-6}$
14	SERPINA1	94842084	94858030	73	17	24294	5.29	$6.3*10^{-8}$	0.0012

Supplementary Figure S1 Gene-based association analysis (MAGMA)

a) – Manhattan plot for individual genetic variants used in the analyses (available in at least 20,000 participants) (red line, genome-wide significance level $p=5*10^{-8}$); **b)** – Manhattan plot for genes used in MAGMA (n=18,814) (red line, Bonferroni adjusted significance level p=0.05); **c)** – gene Q-Q plot for gene-based analysis; **d)** – top seven genes identified in gene-based analysis.

CHR – chromosome; **MAGMA** – Multimarker Analysis of GenoMic Annotation; **N** – lowest number of participants contributing genetic variants for the corresponding gene; $\mathbf{N_{SNPS}}$ – number of genetic SNPs annotated to the gene and used in the analyses; $\mathbf{N_{PARAM}}$ – number of parameters used in the model by MAGMA; **P** – unadjusted p-value calculated by MAGMA for each gene, based on a SNP-wide mean model using user supplied p-values (for all SNPs annotated to the gene); $\mathbf{P_{ADJ}}$ – p-value with Bonferroni correction; **SNP** – single nucleotide polymorphism; **ZSTAT** – Z-value for the gene, calculated by MAGMA based on a permutation p-value, used as the measure of gene association in the gene-based analysis.
